# Adult hippocampal neurogenesis and pattern separation in DG: a role for feedback inhibition in modulating sparseness to govern population-based coding

**DOI:** 10.3389/fnsys.2015.00120

**Published:** 2015-08-20

**Authors:** Kathleen McAvoy, Antoine Besnard, Amar Sahay

**Affiliations:** ^1^Center for Regenerative Medicine, Massachusetts General Hospital, Harvard Medical SchoolBoston, MA, USA; ^2^Harvard Stem Cell Institute, Harvard UniversityCambridge, MA, USA; ^3^Department of Psychiatry, Massachusetts General Hospital, Harvard Medical SchoolBoston, MA, USA

**Keywords:** dentate gyrus, adult hippocampal neurogenesis, pattern separation, interference, discrimination, generalization, sparseness, feed-back inhibition

## Abstract

The dentate gyrus (DG) of mammals harbors neural stem cells that generate new dentate granule cells (DGCs) throughout life. Behavioral studies using the contextual fear discrimination paradigm have found that selectively augmenting or blocking adult hippocampal neurogenesis enhances or impairs discrimination under conditions of high, but not low, interference suggestive of a role in pattern separation. Although contextual discrimination engages population-based coding mechanisms underlying pattern separation such as global remapping in the DG and CA3, how adult hippocampal neurogenesis modulates pattern separation in the DG is poorly understood. Here, we propose a role for adult-born DGCs in re-activation coupled modulation of sparseness through feed-back inhibition to govern global remapping in the DG.

## Introduction

The dentate gyrus (DG) of all mammals harbors neural stem cells that generate new dentate granule cells (DGCs) throughout life (Altman and Das, 1965; Kaplan and Hinds, 1977; Cameron et al., 1993; Kuhn et al., 1996; Eriksson et al., 1998; Seri et al., 2001; Knoth et al., 2010; Spalding et al., 2013). The differentiation of neural stem cells into mature DGCs is marked by changes in physiological properties and connectivity (Zhao et al., 2008; Ming and Song, 2011; Aimone et al., 2014). Whereas young (4–6 weeks old) adult-born DGCs are generally thought to exhibit low input specificity, heightened synaptic plasticity and excitability, mature 6–8 weeks old adult-born DGCs are more similar to developmentally generated DGCs with high input specificity, lower excitability and synaptic plasticity (Schmidt-Hieber et al., 2004; Espósito et al., 2005; Laplagne et al., 2006, 2007; Ge et al., 2007, 2008; Gu et al., 2012). Investigations into the functions of adult hippocampal neurogenesis have suggested numerous roles including the modulation of interference (Becker, 2005; Wiskott et al., 2006; Becker and Wojtowicz, 2007; Garthe et al., 2009; Deng et al., 2010; Sahay et al., 2011a; Burghardt et al., 2012), memory resolution (Aimone et al., 2011), input specific re-activation (Tashiro et al., 2007; Aimone et al., 2011), memory persistence (Arruda-Carvalho et al., 2011; McAvoy et al., 2014; Wang et al., 2014) and forgetting (Chambers et al., 2004; Deisseroth et al., 2004; Weisz and Argibay, 2012; Akers et al., 2014). Behavioral studies using the contextual fear discrimination paradigm have found that selectively augmenting or blocking adult hippocampal neurogenesis enhances or impairs discrimination under conditions of high, but not low, interference (Sahay et al., 2011b; Kheirbek et al., 2012; Nakashiba et al., 2012; Niibori et al., 2012; Tronel et al., 2012). Studies using a delayed non-match to place paradigm and touch screen based object spacing detection assays have obtained similar results in some, but not all studies (Clelland et al., 2009; Pan et al., 2012; Groves et al., 2013; Swan et al., 2014; Zhang et al., 2014). However, the neural mechanisms by which adult hippocampal neurogenesis modulates interference in these tasks are poorly understood (Yassa and Stark, 2011; Piatti et al., 2013; Lepousez et al., 2015; Wadiche and Overstreet-Wadiche, 2015).

Theoretical and experimental studies have posited a critical role for the DG in modulating interference between similar inputs through pattern separation, a process by which similar inputs are made more distinct during storage (McNaughton and Morris, 1987; O’Reilly and McClelland, 1994; Gilbert et al., 2001; Rolls and Kesner, 2006; Bakker et al., 2008; Treves et al., 2008). At a network level, pattern separation in the DG is supported by encoding of similar inputs by differential firing rates of place cells (rate remapping) or the recruitment of non-overlapping ensembles of neurons (or global remapping; Leutgeb et al., 2007; Neunuebel et al., 2013; Neunuebel and Knierim, 2014). Although discrimination of similar contexts (Niibori et al., 2012; Deng et al., 2013; Czerniawski and Guzowski, 2014) elicits global remapping in the DG and CA3 and blockade of adult hippocampal neurogenesis impairs population-based coding in CA3 (Niibori et al., 2012), evidence for how adult-born DGCs contribute to rate remapping and global remapping in the DG is absent. One feature of the DG that has long been recognized as conducive for pattern separation is sparseness of activity (Treves and Rolls, 1992; Jung and McNaughton, 1993; McClelland and Goddard, 1996; Chawla et al., 2005; Colgin et al., 2008; Pernia-Andrade and Jonas, 2014). A sparse coding scheme is thought to support pattern separation by facilitating the recruitment of non-overlapping populations of neurons to encode similar inputs (Treves and Rolls, 1992; McClelland and Goddard, 1996; Colgin et al., 2008; Faghihi and Moustafa, 2015; Petrantonakis and Poirazi, 2015). We recently proposed that adult-born DGCs modulate sparseness of activity in the DG through recruitment of feed-back inhibition to influence pattern separation (Sahay et al., 2011a). We hypothesized that young adult-born DGCs recruit feed-back inhibition via mossy cells and hilar interneurons to dictate sparseness of activity in the DG and this in turn, influences global remapping. Here, we discuss two recent experimental studies that have begun to address this idea and integrate insights gleaned from these studies to formulate a proposal for how adult-born DGCs influence global remapping in the DG.

## Experimental Advances Linking Adult Hippocampal Neurogenesis, Sparseness and Feed-Back Inhibition

As a first step towards addressing how adult hippocampal neurogenesis may facilitate global remapping, we sought to test whether levels of adult hippocampal neurogenesis affect sparseness of activity in the DG (Ikrar et al., 2013). We used voltage sensitive dye imaging (VSDI) in combination with laser photostimulation and electrical stimulation of the granule cell layer (GCL) to visualize depolarization induced spread of the voltage sensitive dye (VSD) signal in *ex vivo* slices from mice in which either adult hippocampal neurogenesis was blocked by targeted x-irradiation or enhanced by genetic deletion of the pro-apoptotic gene Bax in adult neural stem cells. We found that genetically enhancing the number of adult-born DGCs (8 weeks of age and younger) engendered a reduction in spread of VSD signal and strength of neuronal activation in the DG. Conversely, ablating 14 weeks of age and younger adult-born DGCs produced a trend towards increased excitability of the DG. In these experiments the change in the VSD signal was seen throughout the GCL suggesting a non-cell autonomous role for adult-born DGCs in modulating the activation of mature DGCs. Mossy fibers of DGCs synapse onto hilar interneurons and mossy cells, both of which can modulate the activity of the DG. Activation of hilar interneurons and mossy cells is thought to promote local inhibition and long-range activation and inhibition of DGCs, respectively (Freund and Buzsáki, 1996; Scharfman and Myers, 2013; Hu et al., 2014). Changing levels of adult hippocampal neurogenesis did not appear to affect miniature inhibitory post-synaptic currents in the DG; but increasing the number of 4 weeks old adult-born DGCs increased their connectivity with hilar interneurons (Ikrar et al., 2013). Together, these observations demonstrate that levels of adult hippocampal neurogenesis modulate sparseness of activity in the DG as assessed by changes in excitability and suggest a role for excitatory drive onto hilar interneurons as an underlying circuit mechanism (Ikrar et al., 2013). However, whether expanding or ablating populations of adult-born DGCs of various ages differentially affects excitability of the DG was not addressed. In this regard, it is noteworthy to emphasize that a previous report found that genetic ablation of the population of 8 weeks and younger adult-born DGCs decreased sparseness of activity in the DG *in vivo* only under conditions of high interference in memory processing (Burghardt et al., 2012). The use of *ex vivo* slices in our study precluded assessment of the contributions of the commissural-associational pathway that conveys feed-forward excitation and inhibition onto DGCs (Myers and Scharfman, 2009; Jinde et al., 2012; Scharfman and Myers, 2013). Finally, our study did not causally examine whether adult-born DGCs recruit feed-back inhibition to modulate excitability of the DG and whether adult-born DGCs of different ages differentially recruit feed-back inhibition.

Temprana et al. (2015) succeeded in illuminating some of these key unaddressed questions. The authors employed retroviruses to express the light activated channelrhodopsin in adult-born DGCs and examined the impact on activating 4 weeks old or 8 weeks old adult-born DGCs on inhibition of DGCs in slices *ex vivo*. The authors found that pre-activation of 4 weeks old or 8 weeks old adult-born DGCS caused a reduction in spiking in the GCL, but the reduction after pre-activation of 4 weeks old DGCs was half the magnitude of reduction in spiking after pre-activation of 8 weeks old adult-born DGCs. However, chemogenetic activation of 8 weeks old adult-born DGCs, unlike activation of 4 weeks old adult-born DGCs, activated hilar parvalbumin (PV) interneurons *in vivo*. Whether activation of 4 weeks old adult-born DGCs activate other populations of hilar interneurons that mediate feed-back inhibition onto the DG was not addressed. These observations suggest that 4 weeks old adult-born DGCs may exert modest feed-back inhibition through a potentially different mechanism, at least *ex vivo*. As with our study, the use of *ex vivo* slices precluded the possibility of addressing the potentially important contribution of mossy cells to adult-born DGC dependent recruitment of feed-back inhibition *in vivo*. Nevertheless, the findings of this study unequivocally demonstrate that 8 weeks old adult-born DGCs exert significantly greater feed-back inhibition onto the DG than 4 weeks old adult-born DGCs *ex vivo* (Temprana et al., 2015). Whether 8 weeks old adult-born DGCs exert different levels of feed-back inhibition from that recruited by developmentally generated DGCs remains to be addressed. Although specific features of input connectivity and physiology are conserved between 8 weeks old adult-born DGCs and developmentally generated DGCs, not all afferent- and efferent-connectivity of these populations have been systematically characterized. Ontogenic analysis of connectivity of hippocampal neurons has revealed differences in connectivity with interneurons suggesting that 8 weeks old adult-born DGCs and developmentally born DGCs may differ in input specificity and output connectivity (Donato et al., 2015). Importantly, it will be critical to determine whether DGCs of different ages are differentially recruited across stimulation intensities to exert feed-back inhibition and modulate sparseness of activity in the DG *in vivo*. Guided by their data and previous models (Aimone et al., 2011, 2014; Temprana et al., 2015) developed a computational model proposing how increased recruitment of feed-back inhibition coupled with increased input specificity and decreased excitability during maturation, enables 8 weeks old adult-born DGCs to faithfully respond to familiar inputs.

## Model Linking Adult Hippocampal Neurogenesis, Sparseness and Feed-Back Inhibition with Global Remapping in DG

Predictions of how adult hippocampal neurogenesis modulates global remapping in the DG must take into account the extent to which adult-born and developmentally generated DGCs contribute to memory traces or engrams. Cellular and genetic approaches to tag activated neurons during specific epochs of encoding such as during exposure to two similar contexts have found that the vast majority of activated neurons reside in the outer two-thirds of the GCL (Deng et al., 2013; Tronel et al., 2015). Deng and Gage found that DGCs recruited for global remapping during contextual encoding were probably older than 6 weeks of age and largely located where developmentally generated DGCs reside (Altman and Bayer, 1990; Espósito et al., 2005; Laplagne et al., 2006; Mathews et al., 2010; Deng et al., 2013). A second study by Tronel et al. (2015) found that activation of developmentally generated DGCs is more sensitive to changes in context than 14 weeks old adult-born DGCs. Although the contribution of adult-born DGCs at different stages of maturation to global remapping has not been directly examined, post-training ablation and silencing studies suggest that 4 weeks and older adult-born DGCs are part of the contextual memory trace (Arruda-Carvalho et al., 2011; Gu et al., 2012; Wang et al., 2014). Preliminary studies from our laboratory suggest that expanding the population of 5–8 weeks old adult-born DGCs maintains long-term contextual fear memory suggesting a role for this population in maintenance of the contextual memory trace (McAvoy et al., 2014). Together, these observations predict that developmentally generated and mature adult-born DGCs are part of the ensembles recruited in global remapping to generate engrams.

**Figure 1:**
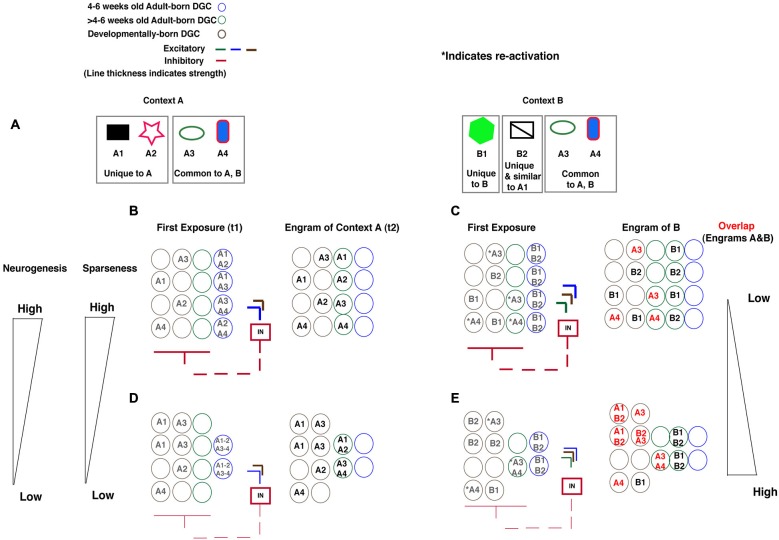
**Model illustrating how levels of adult hippocampal neurogenesis dictate population-based coding in DG to minimize interference between engrams of two similar contexts. (A)** Context A is made up of features that are unique to context A (A1, A2) and common to contexts A and B (A3, A4). Context B is made up of features that are unique to context B (B1, B2) of which B2 is similar to A1. **(B)** High levels of neurogenesis: Exposure to context A (time point t1) activates 4–6 weeks adult born DGCs (blue circles). The activation of these 4–6 weeks adult born DGCs recruits feed-back inhibition to increase sparseness in the DG and promotes encoding of features of context A in developmentally born DGCs (brown circles). The engram for context A is made up of adult-born DGCs that have matured (time point t2) and through Hebbian learning and competition for representation have acquired high input specificity for features of context A (green circles with A1 or A2 or A3 or A4) and developmentally born DGCs that encode features of context A (brown circles with A1 or A2 or A3 or A4). **(C)** High levels of neurogenesis: Exposure to context B re-activates DGCs that have encoded features common to both contexts A and B (Green and brown circles with *A3 or *A4). Re-activation of these DGCs together with activation of 4–6 weeks old adult-born DGCs exerts strong feed-back inhibition onto DG, increases sparseness and promotes encoding of features of context B in individual DGCs. Additionally and importantly, there is lower likelihood that DGCs that have encoded unique features of context A (A1) that are similar to features of context B (B2) are re-activated to encode these features. This results in an engram of context B that has little overlap with engram of context A with the exception of DGCs that encode features common to contexts A and B. **(D)** Low levels of neurogenesis: Exposure to context A (time point t1) activates a small number of 4–6 weeks adult born DGCs (blue circles). The activation of this reduced number of DGCs recruits less feed-back inhibition, decreases sparseness in the DG and promotes encoding of features of A in many more developmentally born DGCs (brown circles). The engram for context A is made up of adult-born DGCs that have matured (time point t2) and have failed to acquired high input specificity for features of context A (green circles with A1–A2 or A3–A4) and developmentally born DGCs that encode features of context A (brown circles). **(E)** Exposure to context B re-activates DGCs that have encoded features common to both contexts A and B (Green and brown circles with *A3 or *A4). Furthermore, because of reduced feed-back inhibition recruited by small number of 4–6 weeks old adult-born DGCs and the re-activated mature adult-born DGCs, sparseness in DG is decreased. This, in turn, increases the likelihood that developmentally born DGCs that have encoded features of context A (A1) that are similar to features of context B (B2) are re-activated. Consequently, the engram of context B has significantly more overlap with engram of context A relative to mice with high levels of adult hippocampal neurogenesis.

Current evidence from studies in mice support differential engagement of feed-back inhibition (Temprana et al., 2015), modulation of DG excitability and sparseness (Ikrar et al., 2013), differences in input specificity (Neunuebel and Knierim, 2012, 2014) and excitability (Schmidt-Hieber et al., 2004; Espósito et al., 2005; Laplagne et al., 2006, 2007; Ge et al., 2007, 2008; Gu et al., 2012) of 4–6 weeks old adult-born DGCs and mature (>6 weeks old) adult-born DGCs). Based on these observations and two recently developed models (Aimone et al., 2011; Kropff et al., 2015; Temprana et al., 2015), we propose that adult-born DGCs contribute to global remapping in DG by expanding the capacity of the DG to encode new information and ensuring high fidelity of re-activation that is coupled with regulation of sparseness through feed-back inhibition. Our model is predicated on two central assumptions. First, the maturation of excitable, immature (4–6 weeks) adult-born DGCs is accompanied by refinement of their synaptic inputs through hebbian learning and competition for neural representation to generate (>4–6 weeks) mature DGCs with high input specificity. The idea is that increasing the number of encoding units available for encoding will promote competition for synaptic inputs and extraction of distinct features to minimize interference (Aimone et al., 2011, 2014; Neunuebel and Knierim, 2012; Kropff et al., 2015; Temprana et al., 2015). Second, limited experimental evidence suggests that adult-born DGCs once mature (>6 weeks of age) are preferentially re-activated by inputs to which they were exposed to when younger (4–6 weeks of age; Tashiro et al., 2007; Aimone et al., 2011). Because the contextual discrimination task in which mice are challenged to discriminate between a training context (Context A) associated with a foot shock and a safe, similar context (Context B; Figure 1A) has been shown to be sensitive to levels of adult hippocampal neurogenesis and involves global remapping in the DG, we will use it to convey our model for how adult-born DGCs contribute to global remapping in the DG. We lead the reader through how changing levels of adult hippocampal neurogenesis affects the engram of context A and context B to dictate the extent of global remapping in the DG (Figure 1).

With high levels of adult hippocampal neurogenesis, features of a novel context (A) are encoded by 4–6 weeks old adult-born DGCs (time point t1). Maturation of this population of 4–6 weeks old adult-born DGCs generates a population of mature adult-born DGCs (>4–6 weeks of age) with high input specificity for features of context A. In addition, feed-back inhibition recruited by 4–6 weeks old adult-born DGCs ensures sparseness of activation in the DG (Figure 1B; time point t2). The engram of context A (time point t2, when 4–6 weeks old adult-born DGCs mature) is made up of adult-born DGCs older than >4–6 weeks of age with high input specificity and developmentally born DGCs. Conversely, with low levels of adult hippocampal neurogenesis, context A is encoded by a smaller pool of 4–6 weeks old adult-born DGCs which mature into DGCs with lower input specificity for features of context A (Figure 1D). Decreased feed-back inhibition (due to a smaller pool of 4–6 weeks old adult-born DGCs) decreases sparseness of the DG and this results in many more developmentally generated DGCs active during encoding of features of context A. Thus, the engrams of context A is made up of adult adult-born DGCs older than >4–6 weeks of age with low input specificity and developmentally born DGCs (Figure 1D, t2).

Upon exposure to a similar context B that shares features with context A (Figure 1A, A3 and A4 are features shared by contexts A and B), developmentally generated and mature (>4–6 weeks of age) adult-born DGCs are re-activated by features shared by contexts A and B. In addition, developmentally generated DGCs that encoded features shared by contexts A and B are also re-activated. With high levels of adult hippocampal neurogenesis there is greater feed-back inhibition recruited by re-activated mature (>4–6 weeks of age) adult-born DGCs and the immature (4–6 weeks old) DGCs that encode novel features of context B than with decreased adult hippocampal neurogenesis (Figures 1C,E). Greater sparseness will promote global remapping since the likelihood of developmentally generated DGCs that have encoded unique features of A to be re-activated by context B will be statistically lower. Thus, levels of adult hippocampal neurogenesis will dictate the overlap in engrams of contexts A and B.

The predictions of this model are readily testable and will necessitate addressing the following questions. First, how does enhancing or silencing populations of adult-born DGCs at distinct stages of maturation or developmentally generated DGCs, impact global remapping in the contextual discrimination paradigm *in vivo*? Second, do adult-born DGCs at distinct stages of maturation or developmentally generated DGCs differentially modulate sparseness and recruit different levels of feed-back inhibition *in vivo*? Third, how are mossy cells and specific populations of interneurons recruited by DGCs to modulate sparseness and global remapping? The complexity of addressing this undertaking is underscored by the fact that engagement of hilar interneurons and mossy cells may be biased by different neuromodulatory systems that innervate these cell types and firing patterns of dentate granule neurons (Freund and Buzsáki, 1996; Torborg et al., 2010). Thus, behavioral state or cognitive demand will dictate the extent to which feed-back inhibition is effectively recruited to modulate sparseness and global remapping. Visualizing ensembles of activated DGCs by calcium imaging *in vivo*, genetic tagging using immediate early genes, cellular compartment analysis of temporal activity by fluorescent *in-situ* hybridization (catFISH), or place cells recordings *in vivo* with concomitant assessment of identity will unequivocally enable direct assessment of DGC re-activation. Together, these approaches will permit resolution of how ablating or expanding populations of adult-born DGCs of different ages influences global remapping in the DG.

## Conflict of Interest Statement

The authors declare that the research was conducted in the absence of any commercial or financial relationships that could be construed as a potential conflict of interest.
